# Hereditary Leiomyomatosis and Renal Cell Cancer (HLRCC) Syndrome

**DOI:** 10.5334/jbsr.3687

**Published:** 2024-09-11

**Authors:** Karel Mercken, Brecht Van Berkel, Liesbeth De Wever

**Affiliations:** 1 Department Radiology, UZ Leuven, campus Gasthuisberg, Leuven, Belgium Herestraat Leuven, Belgium; 2 Department Radiology, UZ Leuven, campus Gasthuisberg, Leuven, Belgium Herestraat Leuven, Belgium; 3 Department Radiology, UZ Leuven, campus Gasthuisberg, Leuven, Belgium Herestraat Leuven, Belgium

**Keywords:** HLRCC, renal cell carcinoma, cutaneous leiomyoma, uterine leiomyoma, fumarate hydratase deficiency

## Abstract

In hereditary leiomyomatosis and renal cell carcinoma syndrome, fumarate hydratase–deficient renal cell carcinomas typically present as aggressive, unilateral, often cystic masses with heterogeneous enhancement. These tumors can metastasize early, making appropriate imaging and staging critical for diagnosis and management.

*Teaching point:* When a renal lesion suspected of RCC is identified in a patient with cutaneous and uterine leiomyomas, HLRCC should be evaluated, which is important for future genetic counseling.

## Introduction

Hereditary leiomyomatosis and renal cell cancer (HLRCC) syndrome is diagnostically challenging due to its variable presentation and clinical features. HLRCC is a rare autosomal-dominant syndrome defined by the triad of cutaneous leiomyomas, uterine leiomyomas, and fumarate hydratase (FH)–deficient renal cell carcinoma (RCC) [1–2]. Germline mutations in the FH gene lead to impaired enzymatic function by altering the Krebs cycle, with accumulation of fumarate. This metabolic disruption fuels tumorigenesis by dysregulating hypoxia-inducible factor pathways, particularly impacting renal tissue, where it drives the development of RCC [3].

Cutaneous leiomyomas may be early indicators of HLRCC, presenting as smooth, hyperpigmented papules or nodules primarily on extensor surfaces, trunk, face, and neck. Uterine leiomyomas are present in 73%–100% of women with FH mutations and may cause symptoms such as menorrhagia and reproductive dysfunction. RCCs occur in 20%–34% of affected families, mainly manifesting as solitary and unilateral type 2 papillary RCC [2].

## Case Report

**Figure 1:**
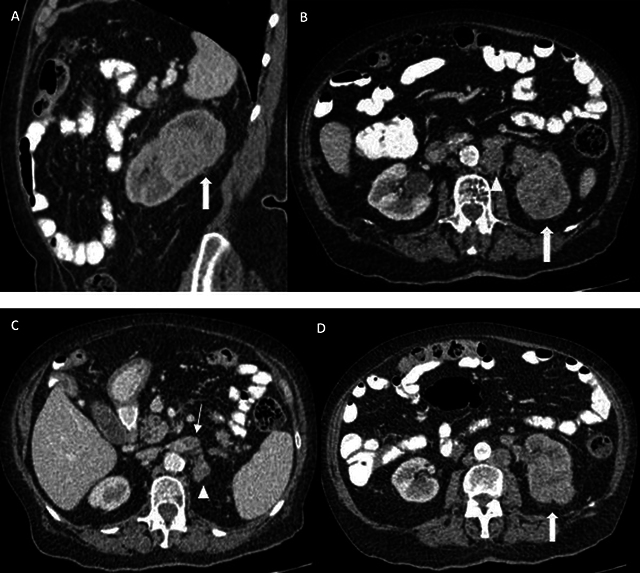
Expansile mass in the upper portion of the left kidney (thick arrows in **A, B, D**), with invasion of the sinusal fat tissue **(B, D)**. A delayed nephrogram is apparent secondary to outflow obstruction due to renal vein thrombosis (thin arrow in **C**). Large, confluent, retroperitoneal lymphadenopathies can be seen (arrowheads in **B, C**).

**Figure 2:**
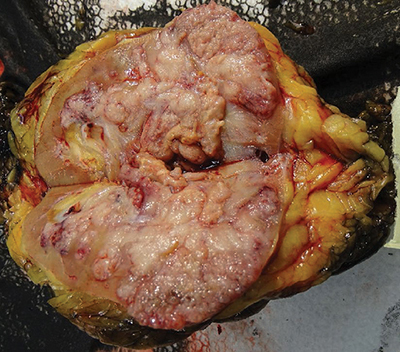
Gross specimen of the resected kidney.

**Figure 3:**
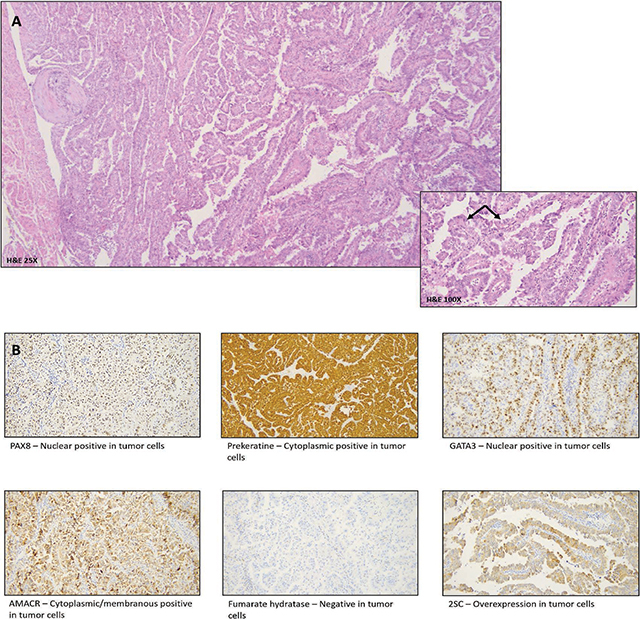
**A.** H&E – Encapsulated tumor with tubulopapillary architecture. Tumor cells show eosinophilic cytoplasm and pleomorphic hyperchromatic nuclei with Fuhrmann nuclear grade 3. **B.** Immunohistochemistry markers with PAX8/panCK positivity.

An 80-year-old female patient presented at the emergency department with painless hematuria. Medical history mentioned the presence of uterine leiomyomas. An abdominal CT showed an expansile mass in the upper pole of the left kidney, with extension in sinusal fat, eccentric necrosis, and tumor thrombus in the left renal vein. Large, confluent retroperitoneal lymph nodes were also present (Figure 1). A radical left nephrectomy was performed. The gross specimen showed a multinodular solid mass, with necrosis and invasion in the sinusal fat and the renal vein (Figure 2). The pathology report mentioned a fibrous, encapsulated tumor, with a trabecular-to-tubulopapillary growth pattern and with large tumor cells that had a predominantly eosinophilic cytoplasm. Tumor cells contained a central, round nucleus and a fine-grained-to-vesicular chromatin pattern with an eosinophilic nucleole. Immunohistochemistry showed absence of FH in the tumor epithelium, indicating a FH-deficient RCC, which additionally supported PAX8+/panCK+ (Figure 3).

**Figure 4:**
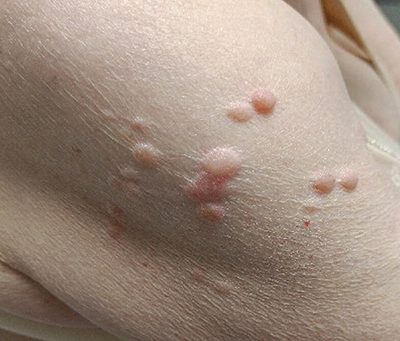
Multiple skin papules/nodules on the upper arm, compatible with cutaneous leiomyoma.

The patient also had multifocal skin lesions, most pronounced on the right arm (Figure 4). These lesions were biopsied, and pathology reported the presence of a cutaneous leiomyoma. Given the history of an FH-deficient RCC, this would fit within HLRCC syndrome.

## Discussion

FH-deficient RCC is a rare and aggressive subtype of renal cancer, resulting from pathogenic mutations in the fumarate hydratase gene. It is characterized by its high malignancy and potential to metastasize even when tumor size is small. Its exact incidence remains unknown [4].

FH-deficient RCC typically presents as a predominantly unilateral and solitary lesion, which distinguishes it from other familial RCC syndromes characterized by bilateral multifocal tumors [5–6]. Tumor size varies considerably, and approximately 50%–80% of cases present with a cystlike component occupying more than 75% of the tumor volume. These cystic formations typically exhibit a polycystic morphology with smooth walls and septa and an eccentric solid component [5]. This aggressive biological behavior results frequently in invasion of the renal sinus fat and hilar collecting system (respectively, in 80% and –50% of cases) as well as capsular and perinephric fat invasion. Renal vein tumor thrombosis is evident in approximately 40% of cases, and extension in the inferior vena cava invasion is also documented [5]. FH-deficient RCC presents with heterogeneous enhancement [5–6]. On DWI-MRI, it presents with diffusion restriction in the solid tumor portions [5–6]. Hemorrhagic changes are observed in approximately 58% of cases [5]. Calcifications may be present in a minority of FH-RCC cases. Metastatic spread in FH-RCC commonly involves retroperitoneal lymph nodes (70%–80%), with liver, bone, mediastinum, lung, adrenal gland, spleen, and breast as other reported sites [5–6].

No specific imaging features reliably distinguish FH-RCC from other RCC subtypes on CT and MRI. Modalities such as magnetic resonance spectroscopy (MRS) might contribute to detection of HLRCC-associated RCC [7].

## Conclusion

In HLRCC syndrome, FH-deficient renal cell carcinomas typically present as aggressive, unilateral, and often-cystic masses with heterogeneous enhancement. They can metastasize even at small size, making early and accurate simaging critical for diagnosis and management.
